# Circulating Anti-Beta2-Glycoprotein I Antibodies Are Associated with Endothelial Dysfunction, Inflammation, and High Nitrite Plasma Levels in Patients with Intermittent Claudication

**DOI:** 10.1155/2013/268079

**Published:** 2013-10-10

**Authors:** Cesar Varela, Joaquin de Haro, Silvia Bleda, Leticia Esparza, Ignacio Lopez de Maturana, Francisco Acin

**Affiliations:** Department of Angiology and Vascular Surgery, Getafe University Hospital, Getafe, 28905 Madrid, Spain

## Abstract

Our aim is to investigate a possible association of circulating anti-beta2-glycoprotein I antibodies (ABGPI) with the endothelial dysfunction, nitric oxide bioactivity dysregulation, and the inflammatory status that surrounds peripheral arterial disease. We carried out an observational translational study, including 50 male patients with intermittent claudication and a healthy control group of 10 male subjects, age and sex matched with the cases. Flow-mediated arterial dilatation (FMAD) was assessed as a surrogate of endothelial dysfunction, and C-reactive protein (hsCRP) was determined as a marker of inflammation. Nitrite plasma levels were measured by colorimetric analysis. Circulating ABGPI titer was detected with indirect immunofluorescence. Titers <1 : 10 represented the reference range and the lower detection limit of the test. Circulating ABGPI titer ≥1 : 10 was detected in 21 (42%) patients and in none of the control subjects (*P* < 0.01). Patients with ABGPI titer ≥1 : 10 had a lower FMAD (*P* = 0.01). The CRP levels were higher in patients with ABGPI titer ≥1 : 10 (*P* = 0.04). The nitrite plasma levels were higher in patients with ABGPI titer ≥1 : 10 (*P* < 0.01). These data suggest that these circulating ABGPI may collaborate in the development of atherosclerosis; however, further prospective studies are required to establish a causal relationship.

## 1. Introduction

The endothelium is responsible for maintaining the balance between the different factors involved in the vascular wall function. In atherosclerosis, this balance is broken, and the endothelium is no longer able to regulate vascular homeostasis. This situation causes endothelial dysfunction characterised by vasospasm, vasoconstriction, local coagulation alterations, abnormal fibrinolysis, and an increase in arterial wall cell proliferation. Endothelial dysfunction acts as a primary pathogenic event, as it occurs before structural change are evident on angiogram or ultrasound scan, and it is not correlated with the disease's severity [1]. The loss of endothelial regulation has been attributed to a reduction in nitric oxide bioactivity and to an increased oxygen-free radical formation in the context of the proinflammatory status found in atherosclerosis [2, 3]. 

On the other hand, there is currently a wide variety of data pointing to a possible autoimmune origin of atherosclerosis [4–11]. This hypothesis is biologically plausible, as chronic vascular inflammation observed in atherosclerosis is based on the dysregulation of the immune system activity. In this context, circulating anti-beta2-glycoprotein I antibodies (ABGPI) have been associated with peripheral arterial disease (PAD) and coronary arterial disease [12–14]. These autoantibodies are directed against beta2-glycoprotein antigens, a plasmatic protein that displays an intense tropism for endothelial cell membrane phospholipids [10, 15–17]. Circulating ABGPI activity involves dendritic cells activation and could interact with endothelial cells through a nuclear factor kappa B (NF-*κ*B) dependent mechanism, leading to the expression of leukocyte adhesion molecules and increasing the release of proinflammatory cytokines [10, 11, 18]. ABGPI also collaborate in the opsonization of apoptotic cells [19] and could disrupt the physiological angiogenic process [20]. 

Data obtained from previous studies suggest that the endothelial dysfunction of PAD patients could be perpetuated in an inflammatory feedback, in which nitric oxide plays a key role [3]. On the other hand, some reports suggest that ABGPI could modulate vascular wall dysfunction inducing vessel smooth cell function deterioration and through the inhibition of endothelial nitric oxide production causing vascular thrombosis [11, 21, 22].

Our aim is to investigate the association of circulating ABGPI with the endothelial dysfunction, nitric oxide bioactivity dysregulation, and the inflammatory status that surrounds PAD in order to explore a possible role of these autoantibodies on the onset and development of atherosclerosis. 

## 2. Material and Methods

An observational translational study was conducted, including male patients with intermittent claudication due to PAD after haemodynamic confirmation of the disease by Doppler and treadmill exercise testing. None of them had been previously revascularized or presented tissue lesions of the lower limbs. The control group included healthy male subjects with normal results on vascular examination and no cardiovascular risk factors, who were not in receipt of any pharmacological treatment, matched by age within two years with PAD patients. The subjects were recruited at the Angiology and Vascular Surgery outpatients clinic. All included individuals signed an informed consent form, according to the principles of the Helsinki Declaration, and the study protocol was approved by our Hospital Ethics Committee. The design of this research was based on the best possible control of the experimental variables in order to achieve results, as reliable as possible, of the pathogenic mechanisms of the disease, using human models within the highest ethical corrections. On the other hand, this study did not pretend to obtain information that could be efficiently applied to the daily clinical practice; therefore, the external validity parameters that are evaluated in clinical trials are not applicable for this research.

All those subjects with documented diagnosis of autoimmune or rheumatologic diseases, transplanted or immunosuppressed and those treated with immunosuppressors or systemic and/or inhaled corticosteroids were excluded. Remaining subjects underwent a serological screening for autoimmune, rheumatologic disease markers and markers for neoplasia (CEA, CA. 125, CA 15.3, and CA 19.9). Antibodies ANA, anti-DNA, anti-LKM, ANCA, antimitochondria, and antismooth muscle were analyzed by means of indirect immunofluorescence and antibodies anti-SM, anti-Jo, anti-LA, anti-Scl 70, antibasement membrane, anti-RO, and anti-RNP were measured using ELISA test. All individuals seropositive to any marker were also excluded from the study. During five months, 462 patients were screened at our outpatients clinic, but only 50 PAD patients and 10 healthy subjects met the inclusion criteria and were finally recruited for the research.

Cardiovascular risk factors and medical treatment were all recorded at inclusion. The study endpoints were measured in all the subjects after fasting for 12 hours (including suspension of their regular medication). A peripheral blood sample was obtained for basic laboratory measurements (glycaemia, electrolytes, and renal function), lipid profile, nitrite plasma levels measurements, highly sensitive C-reactive protein (hsCRP) levels determination, and circulating ABGPI titer detection. Following the blood samples, the flow-mediated arterial dilatation (FMAD) was performed after a 10-minute rest period in supine position. FMAD is an ultrasound test based on the ability of endothelial cells to detect changes in shear stress [3] and is one of the most effective and reliable indirect methods for estimating endothelial dysfunction. Ultrasound examinations were performed by a single observer credited for noninvasive laboratory explorations using a 7.5 MHz linear transducer (Esaote, Technos, Genova, Italy). The visualization of the intima-media interface was optimized by correct adjustment of gain and depth parameters. The measurements were made by an independent observer who was blind to the test's circumstances and the characteristics of the studied subjects.

### 2.1. Determination of Nitrite Plasma Levels

Plasma nitrite concentration was measured by colorimetric analysis using the Griess reaction [23]. This is a chemical reaction which uses sulphanilamide and N-1-naphthylethylenediamine dihydrochloride under acid conditions (phosphoric acid). The system is capable of detecting NO_2_^−^ in a variety of biological and experimental fluids and has a limit of detection of 2.5 *μ*m (125 pmol). Each sample was analyzed in triplicate, and the mean of the three determinations was taken. In previous studies, we have evaluated the reproducibility of the test in the serum sample of 20 patients. The coefficient of variation was < 5% [3]. 

### 2.2. Determination of hsCRP Levels

Highly sensitive C-reactive protein levels were measured using highly sensitive, automated immunoassay (Roche Diagnosis, Basel, Switzerland). This test provides a low detection limit of 0.2 mg/L and a variation coefficient of 4.2% in 4 mg/L and 6.3% in 1 mg/L. Each sample was analyzed in triplicate, and the mean of the three values was used for the analysis. 

### 2.3. Detection of Circulating ABGPI

The titer of circulating antiendothelial cell antibodies was detected by indirect immunofluorescence using a diagnosis reagent kit from EUROIMMUN (Medizinische Labordiagnostika AG, Luebeck, Germany) with a TITERPLANE technique. These autoantibodies require plasmatic beta2-glycoprotein I for endothelial cell binding and cell activation [24]. As previously mentioned, other antibodies capable of interacting with the endothelium were excluded before subjects, final inclusion. Therefore, detected antibodies were, with high probability, circulating ABGPI. Cultivated umbilical vein endothelial cells covered the reaction areas of a biochip. Slides were incubated with patient's diluted serum samples. Positive reaction was marked by granular staining in cytoplasm of human umbilical vein endothelial cells with fluorescein-labeled antibodies and were visualized by fluorescence microscopy. According to the manufacturers, titers of <1 : 10 represent the reference range and the lower detection limit of the test. Patients were classified into “very high autoimmune activity” if they showed a circulating ABGPI titer ≥1 : 100.

### 2.4. Determination of FMAD

The ultrasound transducer was applied proximal to the antecubital fossa, and a longitudinal image of the brachial artery was obtained. Three measurements of the arterial diameter were determined, coinciding with the final diastolic phase of the doppler curve, and the mean value was calculated. A blood pressure cuff was then placed distal to the measurement area and inflated to a pressure of 250 mmHg for five minutes. New measurements of the arterial diameter in the final diastolic phase were obtained, 60 seconds after the cuff was deflated. All the measurements were made in the same environmental conditions. When the blood flow of an arterial segment is occluded, the resulting hypoxia causes vasodilatation of the distal vascular bed, reducing vascular resistance. This situation entails that once the occlusion disappears, blood flow increases. The resulting shear pressure exerted on the endothelium stimulates the expression of endothelial nitric oxide synthase (eNOS), with the resulting release of nitric oxide. Nitric oxide causes vasodilation by relaxing the smooth muscle cells of the vascular wall [25]. FMAD was defined as the difference between baseline and postischemic arterial diameter, regarding with the baseline diameter and expressed as a percentage (postischemic diameter − baseline diameter/baseline diameter × 100 = %). This technique has been previously validated in our laboratory [1]. 

### 2.5. Statistical Analysis and Sample Size

The relationship of circulating ABGPI with endothelial dysfunction (FMAD), inflammation (levels of hsCRP), and nitric oxide metabolism (nitrite plasma levels) was analyzed in order to describe the potential association between these variables. 

Data were processed using the SPSS 15.0 statistical software package. Differences between groups were considered statistically significant for a *P* < 0.05 in two-tailed test. The normality of continuous variables was analyzed using Kolmogorov-Smirnov and Shapiro-Wilk tests. The association between categorical variables was studied using the chi-square test and the Fisher's Exact test when required. The association between continuous variables was analyzed using the Mann-Whitney *U* test. Correlation between continuous variables was measured using the Spearman's *p* test. Categorical variables were expressed as percentage and continuous variables as the median (interquartile range [p25–p75]). All the extreme values and outliers were identified and double checked. 

The statistical power was determined according to a power calculator software available online (http://calculators.stat.ucla.edu/). The number of subjects required to achieve a statistical power of 80% with a bilateral default alpha error of 0.05 was 40 cases.

## 3. Results

Circulating ABGPI titer ≥1 : 10 was detected in 21 [42%] patients. None of the control subjects presented an ABGPI titer ≥1 : 10 (*P* < 0.01). All the antibodies detected were IgG isotype. Twenty-four percent [24%] of the studied patients were classified as “very high autoimmune activity”. The baseline characteristics of the sample are described in Table 1.

### 3.1. Relationship between Circulating ABGPI and FMAD

**Figure 1:**
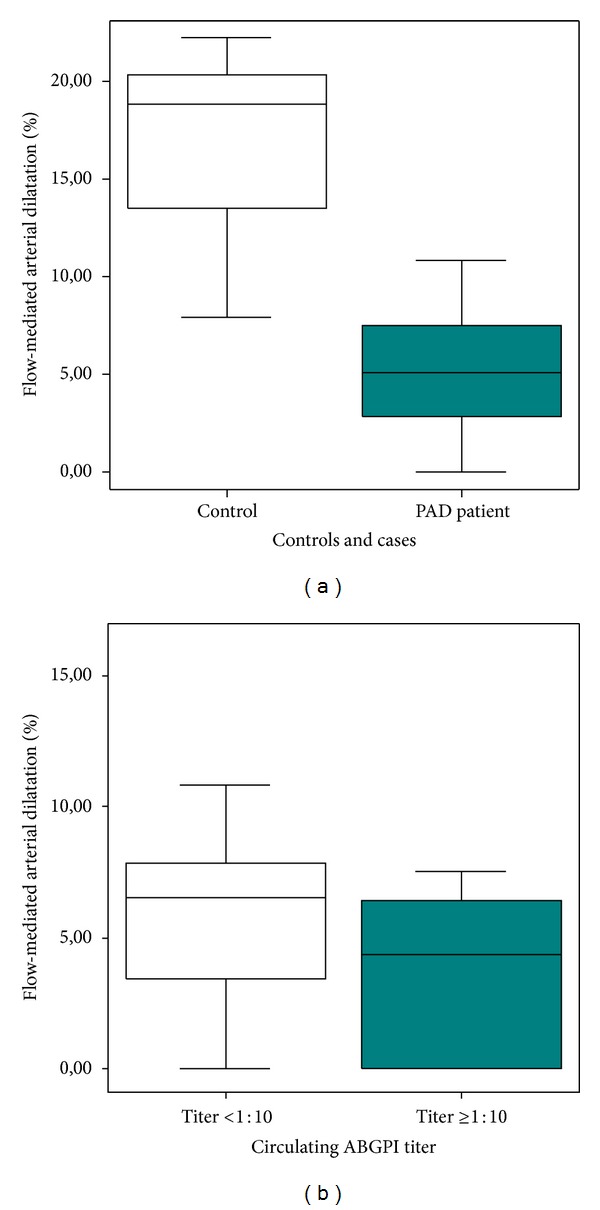
Flow-mediated arterial dilatation (FMAD) results. (a) Peripheral arterial disease patients showed lower FMAD values (*P* < 0.01). (b) FMAD was lower in patients with circulating anti-beta2-glycoprotein I antibodies (ABGPI) titer ≥1 : 10 (*P* = 0.01).

FMAD was lower in PAD patients than in control subjects (5.04 [2.10–7.50] versus 18.80 [12.07–20.81] %, *P* < 0.01). FMAD was also lower in patients with circulating ABGPI titer ≥1 : 10 than in patients with circulating ABGPI titer <1 : 10 (4.34 [0–6.05] versus 6.55 [3.40–7.85] %, *P* = 0.01) (Figure 1). When we analyzed the FMAD according to the stratification by “autoimmune activity”, we observed that patients with “very high autoimmune activity” presented also a lower FMAD (2.17 [0–6.40] versus 5.10 [3.20–7.50] %, *P* = 0.02). 

### 3.2. Relationship between Circulating ABGPI and hsCRP Levels

**Figure 2:**
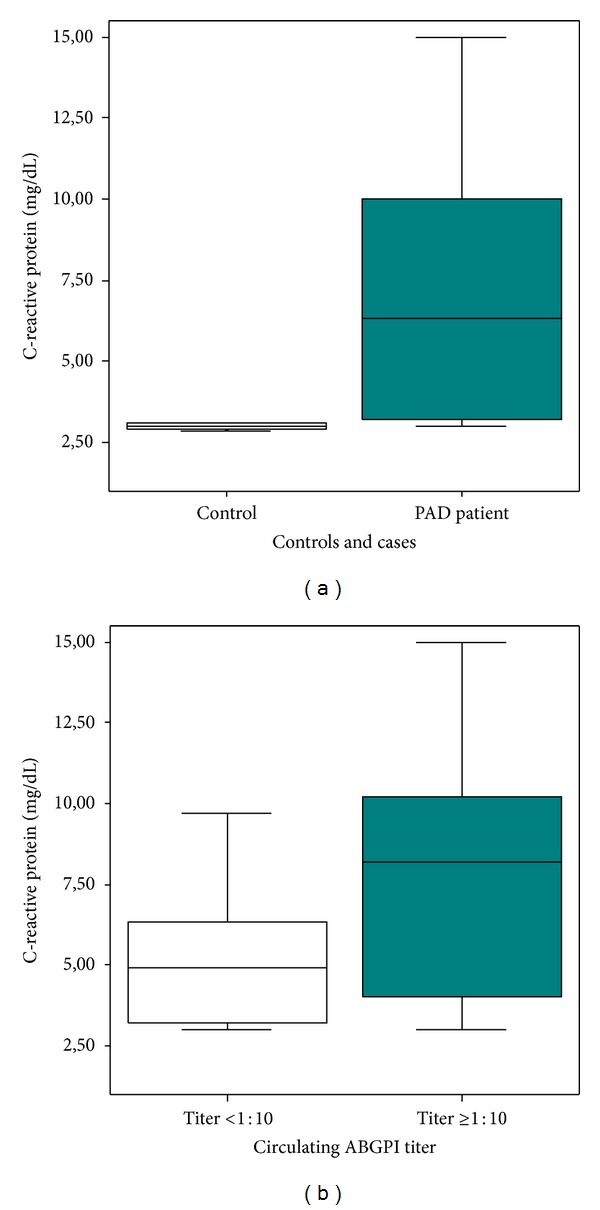
C-reactive protein (hsCRP) results. (a) Peripheral arterial disease patients showed higher hsCRP levels (*P* < 0.01). (b) We observed higher hsCRP levels in patients with circulating anti-beta2-glycoprotein I antibodies (ABGPI) titer ≥1 : 10 (*P* = 0.04).

**Figure 3:**
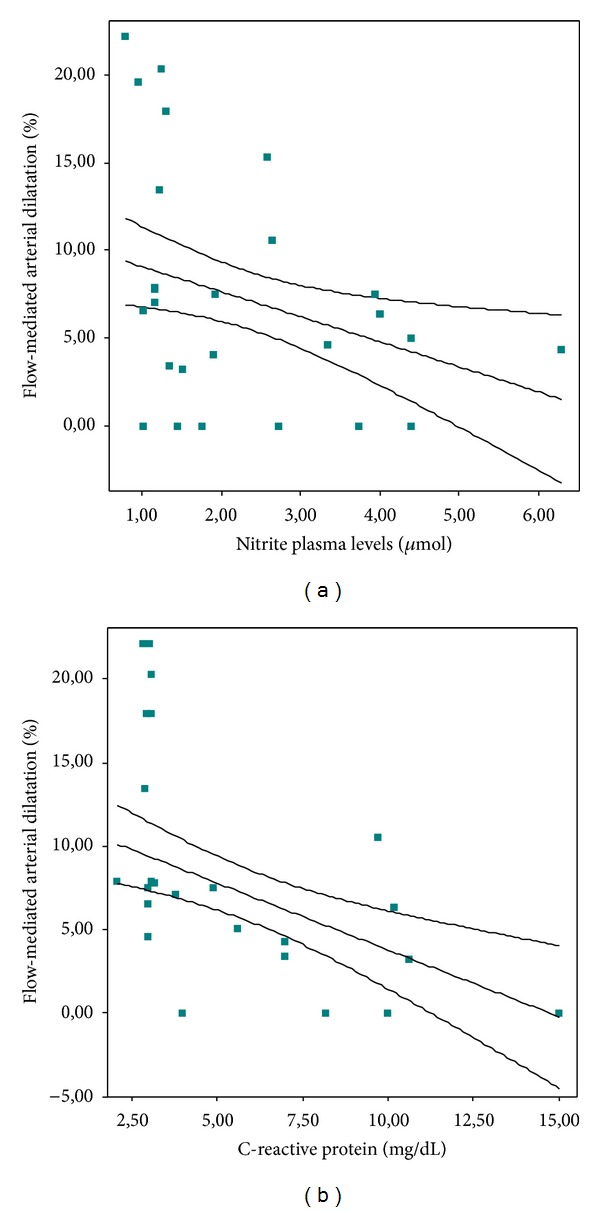
Relationship of flow-mediated arterial dilatation (FMAD) with nitrite plasma levels and C-reactive protein (hsCRP) levels. (a) Trend towards a negative correlation between FMAD and nitrite plasma levels (*r* = −0.24, *P* = 0.07). (b) Negative correlation between FMAD and hsCRP (*r* = −0.63, *P* < 0.01).

Highly sensitive C-reactive protein levels were higher in PAD patients than in control subjects (6.30 [3.20–10.00] versus 3.01 [2.86–3.10] mg/dL, *P* < 0.01). We found higher hsCRP levels in patients with circulating ABGPI titer ≥1 : 10 than in patients with circulating ABGPI titer <1 : 10 (8.20 [3.50–10.20] versus 4.90 [3.20–7.00] mg/dL, *P* = 0.04) (Figure 2). Moreover, hsCRP levels were higher in “very high autoimmune activity” patients (9.20 [7.00–10.20] versus 4.35 [3.00–9.02] mg/dL, *P* < 0.01). We found a negative correlation between hsCRP levels and FMAD (*r* = −0.61, *P* < 0.01) (Figure 3). 

### 3.3. Relationship between Circulating ABGPI and Nitrite Plasma Levels

**Figure 4:**
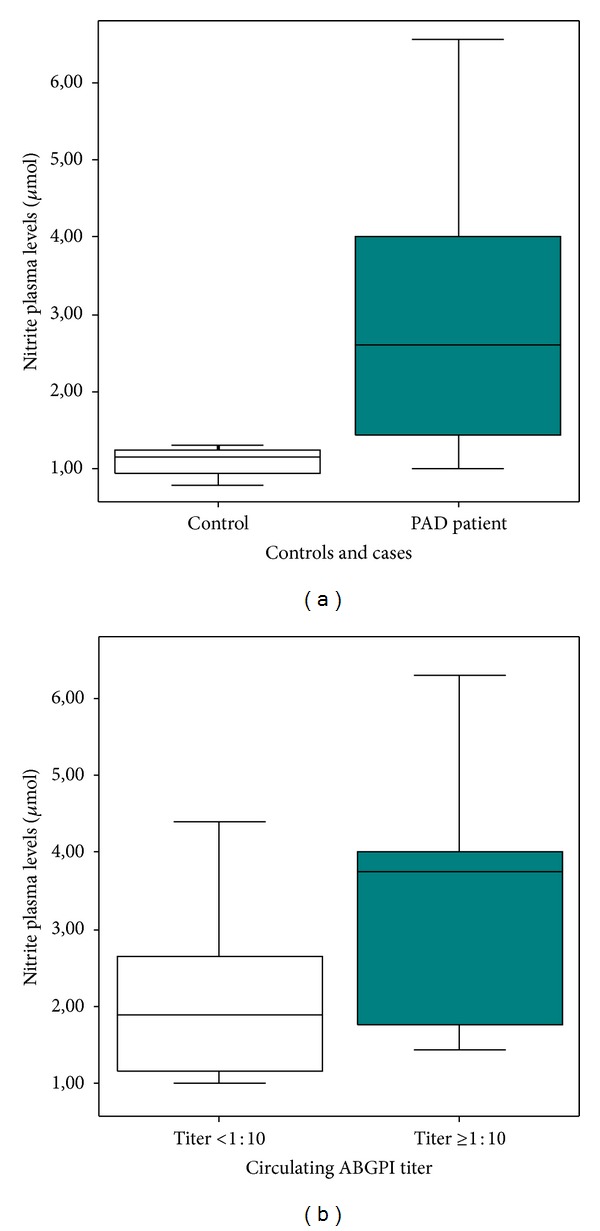
Nitrite plasma levels. (a) Peripheral arterial disease patients showed nitrite plasma levels (*P* < 0.01). (b) Nitrite plasma levels were higher in patients with circulating anti-beta2-glycoprotein antibodies (ABGPI) titer ≥1 : 10 (*P* < 0.01).

Nitrite plasma levels were higher in PAD patients than in control subjects (2.61 [1.41–4.00] versus 1.16 [0.91–1.26] *μ*M, *P* < 0.01). Nitrite plasma levels were also higher in patients with circulating ABGPI titer ≥1 : 10 than in patients with circulating ABGPI titer <1 : 10 (3.74 [1.75–4.00] versus 1.90 [1.16–3.52] *μ*M, *P* < 0.01) (Figure 4). Nitrite plasma levels were higher in “very high autoimmune activity” patients (3.87 [1.75–4.00] versus 2.26 [1.17–3.93] *μ*M, *P* < 0.01). We found a trend towards a negative correlation between nitrite plasma levels and FMAD (*r* = −0.24, *P* = 0.07) (Figure 3). 

## 4. Discussion

This study shows an association between circulating ABGPI and the endothelial dysfunction and inflammatory status that surrounds PAD. Patients with high titer of this autoantibody showed a lower FMAD and higher nitrite plasma levels. The observed correlation between these two variables increases the consistency of our results and suggests that nitric oxide pathway could be involved in the events triggered by ABGPI in the development of the endothelial dysfunction of PAD patients. 

Nitric oxide is the principal vasodilator released by the endothelium and is synthesized in endothelial cells as a result of the enzymatic activity of eNOS, which is continuously expressed [22, 25]. The loss of the molecule's metabolism homeostasis could be one of the main factors that triggers endothelial dysfunction in the early stages of atherosclerosis [2]. Circulating ABGPI could disrupt nitric oxide metabolism through eNOS activity inhibition [22, 26]. According to experimental data, these autoantibodies could antagonize the function of eNOS through ApoER2 endothelial receptors decreasing the bioavailability of nitric oxide and inducing an increase of leukocyte adhesion and thrombosis [22, 26]. In this sense, the role of beta2-glycoprotein I in ABGPI antagonism of eNOS has been measured by loss-of-function experiments comparing the actions of these antibodies in the presence or absence of their specific antigen on the endothelial cell surface. When cells were deprived of beta2-glycoprotein I, ABGPI did not cause eNOS inhibition, indicating that this plasmatic protein is required for the ABGPI action [22].

 However, the proinflammatory situation detected in PAD patients could by itself promote a nitric oxide metabolism disruption and, hence, endothelial dysfunction. The nitric oxide activity is the result of the balance between its production by NOS and its inactivation by oxygen free radicals. In this sense, there is an inducible isoform of NOS (iNOS), which is stimulated by cytokines and produces much larger quantities of nitric oxide than other isoforms [27]. CRP could also stimulate the production of nitric oxide by iNOS [28]. In order to work, these enzymes require several cofactors, including tetrahydrobiopterin (BH4) and NADPH. The cofactor BH4 could be inactivated via the oxidative stress impairment seen in an inflammatory media. When BH4 levels are low, the NOS “uncouples” and behaves like an NADPH oxidase, increasing the production of superoxide anion and hydrogen peroxide and reducing the synthesis of nitric oxide. The net balance is therefore a reduction in nitric oxide activity [27, 29]. These free radicals are able also to directly inactivate nitric oxide, producing peroxynitrite, which is cytotoxic, proinflammatory, and a powerful oxidant that may contribute to endothelial damage and the oxidation of lipoproteins of atherosclerotic lesions [29, 30]. Therefore, in a proinflammatory status, nitric oxide production could be enhanced and rapidly inactivated by the oxidative stress resulting in an increase of nitric oxide degradation product levels as nitrites. In this context, we observed higher nitrite plasma levels and lower FMAD values in patients with high circulating ABGPI titers. These patients also showed higher hsCRP levels. These data suggest that circulating ABGPI of PAD patients may induce endothelial dysfunction through a proinflammatory-dependent nitric oxide metabolism dysregulation. Accordingly, the finding of higher nitrite plasma levels and lower FMAD values in PAD patients is congruent with other published data and may be also explained by the chronic proinflammatory status observed in atherosclerosis [3, 19, 31].

It is nowadays commonly accepted that atherosclerosis is a systemic inflammatory vascular disease. In this context, CRP is a systemic marker of inflammation that is moderately elevated in PAD patients. Data obtained from previous studies show that the clinical severity of PAD keeps a linear association with hsCRP plasma levels [3]. High systemic concentrations of CRP have been also associated with the potential development of atherothrombotic events both in patients with known cardiovascular disease and in apparently healthy subjects [31]. This oxidative stress environment is one of the major factors causing protein structural modifications and could induce the appearance of beta2-glycoprotein antigen neocryptic epitopes capable to stimulate ABGPI production. Oxidized beta2-glycoprotein is also able to modulate a phenotypic and functional maturation of dendritic cells which represents the link between innate and adaptive immunity [32]. On the other hand, circulating ABGPI trigger an endothelial cell inflammatory activation through a NF-*κ*B-dependent mechanism, leading to the expression of leukocyte adhesion molecules and increasing the release of proinflammatory cytokines in vitro [18]. Therefore, our results support the findings of these studies as we have found an association between circulating ABGPI titer and hsCRP levels in PAD patients. Moreover, higher hsCRP levels found in our sample of PAD patients are in agreement with the results of previous studies on this issue [3, 19]. As stated previously, CRP disturbs by itself the nitric oxide metabolism [28], situation that induces endothelial dysfunction [19]. Our results support this pathway as we found a trend towards a negative correlation between hsCRP and FMAD values. Hence, circulating ABGPI may collaborate with the chronic proinflammatory status and endothelial dysfunction of PAD patients.

Circulating ABGPI are directed against beta2-glycoprotein antigens. This plasmatic protein displays an intense tropism for endothelial cell membrane phospholipids [15–17]. It has been suggested that antibeta2-glycoprotein I antibodies recognise their antigen bound to the cell membrane, causing endothelial cell damage [10, 18]. In fact, in inflammatory conditions such as atherosclerosis, early endothelial injury could lead to the exposure of phospholipids to the outer cell surface promoting ABGPI formation [10]. Some experimental evidence suggests that apoptotic cells opsonized by ABGPI are preferentially internalized by dendritic cells [10]. It has also been reported that these autoantibodies are able to trigger an endothelial signalling pathway comparable to that used by the IL-1R-Toll-like receptor superfamily [33–35]. These receptors are a key component of the innate immune response against infection and can induce an endothelial inflammatory phenotype after their interaction with specific ligands. Molecular mimetism of these glycoproteins with microbial antigens could justify a Toll-like-receptor-dependent inflammatory response when ABGPI antibodies are present. Circulating ABGPI have also been related with other endothelial membrane receptors like ApoER2 [26]. Future studies on circulating ABGPI endothelial signalling pathway could be of great interest if the relationship of this autoantibody with atherosclerosis is confirmed. The design of ABGPI pathway blockage therapeutic strategies may have a role in atherosclerosis treatment in the future [10, 36, 37]. In this sense, it has been shown that dendritic cells treated with interleukin-10 and growth factor B-1 decreased proliferation of beta2-glycoprotein I-specific effectors/memory CD4 T-cells in vitro [10]. In a recent research, a novel peptide called EMBI has been reported that is based in the fifth domain of the beta2-glycoprotein molecule sequence that bears the phospholipid combined with membrane binding sites. The EMBI peptide is capable of reducing the binding affinity of purified ABGPI to human umbilical vein endothelial cells and penetrates into these cells reducing endothelial cells activity [37]. 

## 5. Conclusions

**Figure 5:**
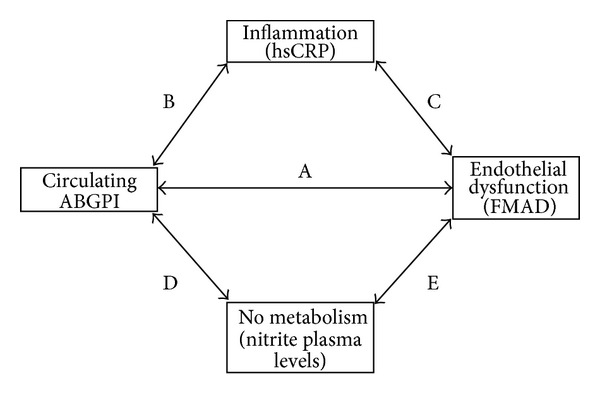
(A) Circulating anti-beta2-glycoprotein antibodies (ABGPI) titer was associated with flow-mediated arterial dilatation (FMAD) values (*P* = 0.01). (B) Circulating ABGPI titer was associated with C-reactive protein (hsCRP) levels (*P* = 0.04). (C) hsCRP levels were correlated with FMAD values (*P* < 0.01). (D) Circulating ABGPI titer was associated with nitrite plasma levels (*P* < 0.01). (E) We found a trend towards a correlation between nitrite plasma levels and FMAD (*P* = 0.07).

This study has some limitations. The design of the research does not allow us to establish a causal relationship between the analyzed variables. The type of test used for circulating ABGPI detection is not as accurate as an ELISA test. However, our findings are consistent with the hypothesis that autoimmunity could play an important role in the genesis and development of PAD. We have found that circulating ABGPI of PAD patients with no previous autoimmune disease are associated to high hsCRP levels, high nitrite plasma levels, and endothelial dysfunction (Figure 5). The pathogenic mechanisms of these autoantibodies make these associations biologically feasible; however, future prospective studies are required to establish a causal relationship. 

## Figures and Tables

**Table 1:** Baseline characteristics of peripheral arterial disease patients according to circulating anti-beta2-glycoprotein I antibodies titer.

	ABGPI titer ≥1 : 10% (*n* = 21)	ABGPI titer <1 : 10% (*n* = 29)	*P* value
Age and cardiovascular risk factors			
Age (years)	65 [60–67]	66 [61–71]	0.58
Diabetes mellitus	6 [29%]	7 [24%]	0.72
History of Smoking	9 [43%]	12 [41%]	0.91
Coronary heart disease	3 [14%]	7 [24%]	0.48
Hypertension	12 [57%]	18 [62%]	0.72
Hyperlipidemia	14 [68%]	16 [55%]	0.41
Cerebrovascular disease	3 [14%]	2 [7%]	0.63
Chronic renal failure	2 [9%]	1 [3%]	0.56
Chronic pulmonary disease	1 [5%]	2 [7%]	1

Medical treatment			
Antiplatelet	13 [62%]	20 [69%]	0.60
Statins	9 [43%]	6 [21%]	0.91
ACE inhibitors	11 [52%]	15 [52%]	0.96
Nitrates	1 [5%]	5 [17%]	0.38
Beta-blockers	2 [9%]	4 [14%]	1
Calcium antagonist	5 [24%]	10 [34%]	0.41
Beta-agonists	2 [9%]	2 [7%]	1
Oral anticoagulants	0 [0%]	3 [10%]	0.25

ABGPI: Anti-beta2-glycoprotein I antibodies.
